# Robotics in Spine Surgery: A Technical Overview and Review of Key Concepts

**DOI:** 10.3389/fsurg.2021.578674

**Published:** 2021-02-23

**Authors:** S. Harrison Farber, Mark A. Pacult, Jakub Godzik, Corey T. Walker, Jay D. Turner, Randall W. Porter, Juan S. Uribe

**Affiliations:** Department of Neurosurgery, Barrow Neurological Institute, St. Joseph's Hospital and Medical Center, Phoenix, AZ, United States

**Keywords:** minimally invasive spine surgery, neuronavigation, pedicle screw fixation, robotics, robotic spine surgery

## Abstract

The use of robotic systems to aid in surgical procedures has greatly increased over the past decade. Fields such as general surgery, urology, and gynecology have widely adopted robotic surgery as part of everyday practice. The use of robotic systems in the field of spine surgery has recently begun to be explored. Surgical procedures involving the spine often require fixation via pedicle screw placement, which is a task that may be augmented by the use of robotic technology. There is little margin for error with pedicle screw placement, because screw malposition may lead to serious complications, such as neurologic or vascular injury. Robotic systems must provide a degree of accuracy comparable to that of already-established methods of screw placement, including free-hand, fluoroscopically assisted, and computed tomography–assisted screw placement. In the past several years, reports have cataloged early results that show the robotic systems are associated with equivalent accuracy and decreased radiation exposure compared with other methods of screw placement. However, the literature is still lacking with regard to long-term outcomes with these systems. This report provides a technical overview of robotics in spine surgery based on experience at a single institution using the ExcelsiusGPS (Globus Medical; Audobon, PA, USA) robotic system for pedicle screw fixation. The current state of the field with regard to salient issues in robotics and future directions for robotics in spinal surgery are also discussed.

## Introduction

In the past decade, the role of robotic systems in surgical fields has expanded, and innovations have flourished (1). Early adopters of this technology have included specialties such as general surgery, urology, and gynecology, where robotics have augmented the ability to manipulate tissue in body cavities (2–4). Early incorporation of robotic systems in these fields has spurred innovation and led to their use in other surgical subspecialties; more recently, robotic systems have been introduced to the field of spine surgery (5).

Treatment of spinal pathologies often requires fixation via the placement of pedicle screws. Techniques for pedicle screw placement were first described in the late 1950s, and since that time, they have undergone a wealth of adaptation and methodological advances. These advances include the description of open and percutaneous approaches using a variety of navigated techniques (6–11). Pedicle screw placement has emerged a prime area of opportunity for the inclusion of robotics in spine surgery. Pedicle screw malposition can lead to serious adverse neurovascular complications, which can contribute to poor outcomes and require reoperation. Accurate screw placement is therefore fundamental to reducing possible iatrogenic complications and improving surgical outcomes.

A key determinant of the widespread adoption of robotics in spine surgery is efficient and accurate screw placement. The accuracy of robotic systems must be similar to or better than that of well-established methods of pedicle screw placement, including free-hand, fluoroscopically assisted, or computer tomography (CT)–assisted screw placement. These systems offer the theoretical advantage of automating inherently repetitive tasks that are subject to human error. Early reports of the use of robotic technologies in spine surgery have shown equivalent accuracy compared with other methods of screw placement (11). Multiple robotic systems have been approved by the U.S. Food and Drug Administration for use. The most current technologies include the ExcelsiusGPS (Globus Medical, Audubon, PA, USA), Mazor X Stealth Edition (Medtronic, Dublin, Ireland), and the ROSA ONE Spine (Zimmer Biomet, Warsaw, IN, USA). These three systems are all now commercially available (12). Unfortunately, direct comparisons of screw placement accuracy between these systems are difficult because of the significant cost and time associated with their adoption.

Herein, we provide a technical overview of the incorporation of robotics in spine surgery based on our institutional experience using the ExcelsiusGPS robotic system for pedicle screw fixation. We also discuss salient issues regarding this technology based on our experience and consider future directions for robotics in spinal surgery.

## Operative Technique

Exact operative technique and surgical workflow will vary on the basis of the specific robotic system that is used. This section will discuss the use of the ExcelsiusGPS system (Globus Medical; Audobon, PA, USA). Robotic systems may be considered for any spinal fusion procedure with planned placement of pedicle screw fixation. At our institution (Barrow Neurological Institute, Phoenix, AZ, USA), robotics have most commonly been incorporated into lumbar fusion procedures, including anterior lumbar interbody fusion, lateral lumbar interbody fusion (13), and transforaminal lumbar interbody fusion (14). Percutaneous pedicle screw fixation has been the most common technique, although open screw placement has been performed as well. Additionally, patients may be placed in the prone or lateral position, depending on the procedure being performed.

**Figure 1:**
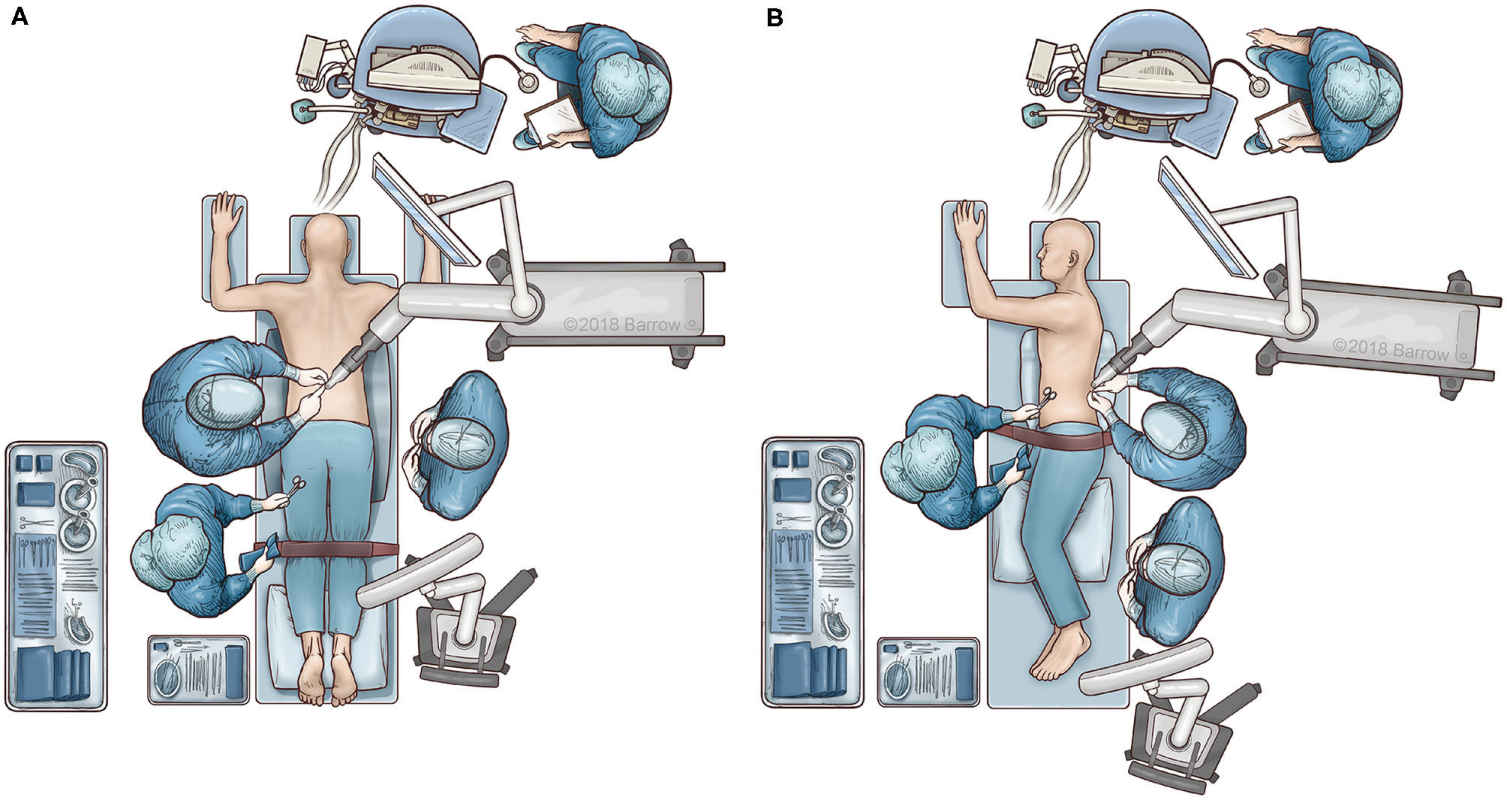
Illustration of surgical workflow. The patient is placed in either a prone or lateral decubitus position. In both positions, the robotic system is placed opposite to the scrub table to simplify draping and ease of access. **(A)** In the prone position, the surgeon stands opposite to the robot. **(B)** In the lateral position, the surgeon stands on the same side as the robotic arm. Used with permission from Barrow Neurological Institute, Phoenix, Arizona.

**Figure 2:**
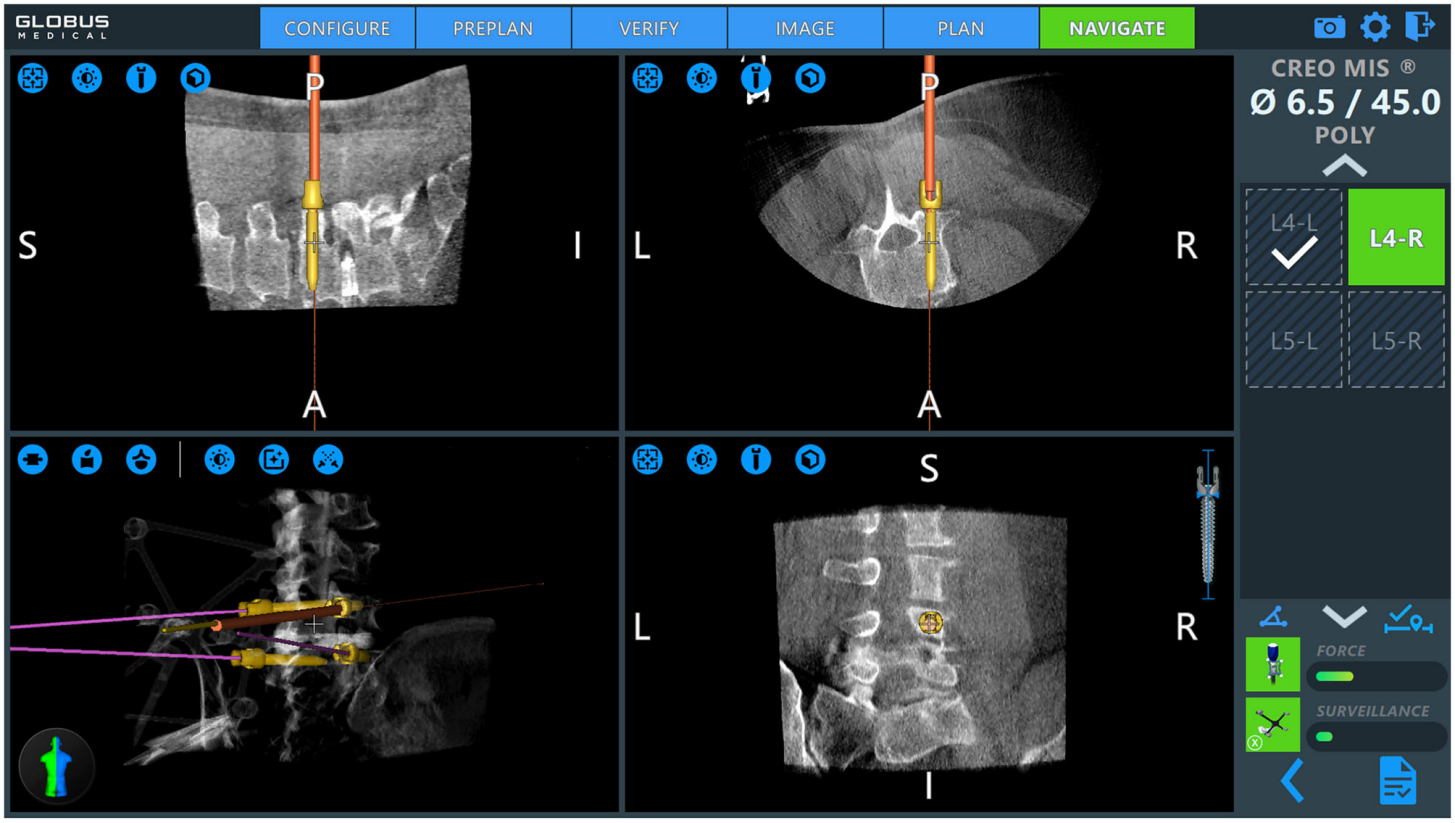
Intraoperative pedicle screw placement using the ExcelsiusGPS robotics system (Globus Medical; Audobon, PA, USA). Screw placement is demonstrated for a right-sided L4 pedicle screw. Used with permission from Barrow Neurological Institute, Phoenix, Arizona.

Patient positioning and operating room set up are shown in Figure 1. The patient is prepped and draped in the usual sterile fashion. First, two small incisions are made over the posterior superior iliac spine bilaterally. The dynamic reference base array and the surveillance marker are then affixed to the posterior superior iliac spine bilaterally. These markers are positioned with a superolateral trajectory. The intraoperative CT registration fixture is then attached to the dynamic reference base array. An intraoperative CT scan using O-arm (Medtronic; Dublin, Ireland) is then performed and is coregistered to the patient's preoperative imaging. A trajectory plan may then be created for each pedicle screw. Alternatively, screw trajectories may be preplanned before the procedure using a preoperative CT scan to decrease intraoperative time. Screw plans may be adjusted and confirmed at this point (Figure 2). The robotic end effector arm then moves into position to guide all movements along this planned trajectory. All subsequent steps can be performed through the end effector arm.

**Figure 3:**
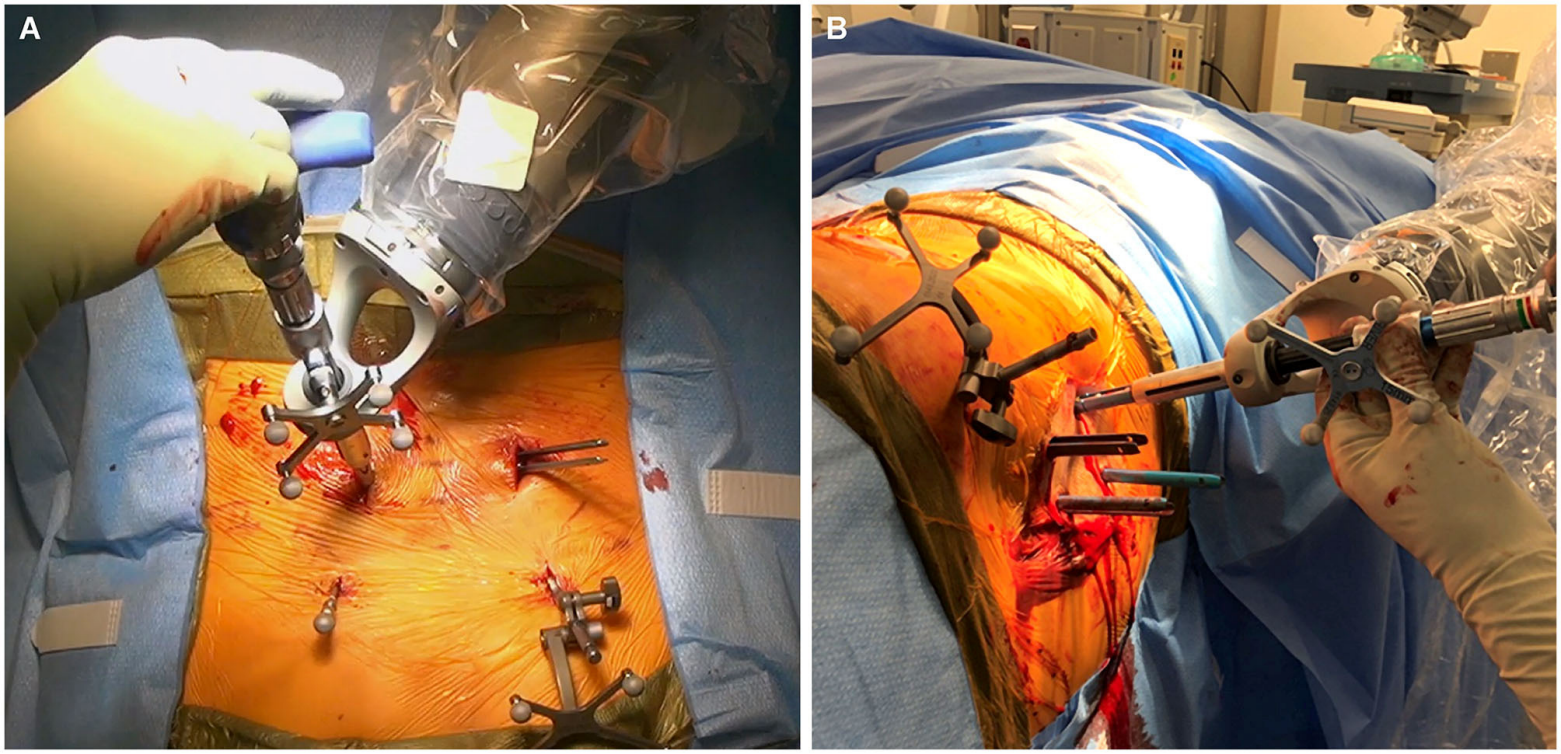
Intraoperative images obtained during robotically assisted pedicle screw placement. **(A)** Pedicle screw placement in the prone position. The dynamic reference base array and surveillance marker are attached to the posterior superior iliac spine bilaterally. **(B)** Pedicle screw placement in the lateral position. Used with permission from Barrow Neurological Institute, Phoenix, Arizona.

First, a stab skin incision is made. The bovie electrocautery is used to dissect through the subcutaneous tissue and the fascia. Importantly, the fascia should be excised medial to the skin incision to allow for the appropriate trajectory toward the screw entry point. The bur is then placed on bone at the screw entry point to create a pilot hole for screw placement. This step is important to prevent skiving of the drill off of the cortical bone and facilitate smooth entry into the cancellous channel. Tapping is then performed under navigation for comparison with the planned trajectory. Finally, the pedicle screw is placed under navigation and is also compared with the planned trajectory (Figure 3A). Once the pedicle screw is in place, the software notifies the surgeon of correct positioning. For each step, a force meter confirms that an appropriate amount of force is being placed on the instruments. The accuracy of screw placement is usually confirmed with a postplacement intraoperative CT scan and may be compared with the planned trajectory.

## Other Surgical Considerations

In our experience, screws have been most commonly placed in a percutaneous fashion. However, open screw placement is also done in appropriate scenarios, such as when accompanied by a spinal decompression for degenerative pathologies. In an open procedure, the dynamic reference base array and the intraoperative CT registration fixture are placed on a spinous process above and below the operative segments rather than on the posterior superior iliac spine. All subsequent steps may be performed in the fashion described above, with care taken not to disrupt the dynamic reference base array within the operative field. In the open exposure, the screw entry site may be directly visualized, and cortical bone may be drilled away to facilitate screw placement.

The above-described technique is for patients who are placed in the traditional prone position. More recently, there has been increasing interest in performing single-position surgery in the lateral position for lumbar fusion, including both anterior lumbar interbody fusion and lateral lumbar interbody fusion, accompanied by pedicle screw fixation. We have used the robotic system for screw placement in this position as well (Figure 3B). In these procedures, the interbody is placed initially through either an anterior or a lateral incision. Pedicle screw fixation is then performed. In the lateral position, the down-side screw trajectory should be modified to decrease medialization of the screw. This modification helps to maintain sterility during placement of down-side screws. Sequential steps are otherwise unchanged.

## Pedicle Screw Placement Accuracy

Widespread adoption of robotic systems for pedicle screw placement must entail safe and reliable accuracy. The most common classification system used in the literature is the Gertzbein-Robbins classification (15). The reported accuracy of pedicle screw placement using robotic systems has generally been high, with rates as high as 94%–98% (16–21). Current literature is mixed regarding the accuracy of robotically placed pedicle screws compared with traditional open freehand techniques. Some studies have reported inferior accuracy with robotically assisted pedicle screw placement. One randomized controlled trial found that 93% of pedicle screws placed with the freehand technique were Gertzbein-Robbins A or B compared with 85% for those placed with the ROSA SpineAssist robot (22).

However, several studies have since reported non-inferiority or superiority of robotically assisted pedicle screw placement. A meta-analysis that included 10 studies found robotically assisted pedicle screw placement performed better than freehand screw placement in terms of “perfect accuracy” (odds ratio 95% confidence interval: 1.38–2.07; *P* < 0.01) as well as “clinically acceptable” (odds ratio 95% confidence interval: 1.17–2.08; *P* < 0.01) (23). Two other meta-analyses reported similar results, showing increased accuracy of robotically assisted pedicle screw placement compared with freehand screw placement (11, 24). A more recent meta-analysis that included nine randomized controlled trials with a total of 696 patients also found the accuracy of pedicle screw placement to be higher with use of robotic systems than with freehand techniques, although results varied on the basis of the different robotic systems that were used (25).

Another technique for pedicle screw fixation that has been widely adopted includes use of navigation with intraoperative CT scan. Some surgeons argue that accuracy of screw placement with use of navigation is high enough that they would not need the use of a robotic arm. Based on our experience, we support the use of a robotic arm for multiple reasons. First, with current technology a CT scan may now be performed pre-operatively. Screw planning is done prior to surgery and this can significantly decrease surgical time compared to the use of intraoperative CT scanning. The ability to plan the screw trajectory ahead of surgery maximizes the fidelity of screw placement. Important considerations such as screw size, length, trajectory, and avoidance of the superior facet are all addressed prior to skin incision. Second, the use of the robotic arm mitigates the human error that is ever present in repetitive manual tasks. Lastly, the authors' experience has been that pedicle screw placement using the robotic arm is less taxing physically for the surgeon.

## Operative Time

Another important factor to consider with regard to the incorporation of robotics into spine surgery is the additional operative time required to use this technology. Multiple studies have found that increased operative times were associated with robotic systems. A commonly considered factor that may contribute to this phenomenon is the steep learning curve for use of this technology. The learning curve associated with robotics has been documented previously by multiple authors who have shown improved accuracy after an initial learning period (26, 27). More persistent exposure to these systems and continued improvements in technology will likely continue to decrease the operative time for these procedures.

## Radiation Exposure

A purported advantage of robotic systems is decreased radiation exposure. The surgeon is not exposed to the initial (preoperative or intraoperative) CT. Minimal intraoperative radiation is then required to register the robotic system to this scan using fluoroscopy. When intraoperative CT is used, no additional fluoroscopy is required for registration. Decreased radiation exposure to the surgeon during robotic procedures has been validated (28). Other studies have shown that decreased overall and per-screw radiation exposure times are associated with robotic systems (29, 30). One randomized controlled trial found that radiation exposure to the surgeon was 10 times lower during robotic procedures compared with fluoroscopy-guided screw placement (31).

## Future Directions

The use of robotic systems in spine surgery is rapidly evolving. Continued adaptation will be important for future expansion in this field. These adaptations should include improvements in efficiency and surgical workflow to facilitate widespread adoption of these systems. Such improvements would involve enhanced imaging software to aid with patient registration, to minimize error, and to assist with trajectory planning both preoperatively and intraoperatively. The aim of robotic systems is to automate repetitive tasks that are subject to alteration and human error. As automation becomes more standardized, this may lead to more uniform patient outcomes. The indications for use of robotic systems will likely continue to expand. Currently, most literature involves fixation of the thoracolumbar spine. Both cervical and pelvic fixation may incorporate robotic systems in the future. In addition, applications for robotics may expand to include more complex spinal procedures, such as decompression, resection of neoplastic lesions, and complex deformity procedures. With more widespread use, head-to-head investigations that compare various robotic systems may help delineate the relative strengths and weaknesses of each system. Finally, the economic viability of these systems should continue to be addressed. Their use will remain limited to resource-rich settings if costs remain very high.

## Conclusions

Robotic systems have been widely adopted throughout the United States and in various surgical subspecialties. This innovative technology continues to permeate the field of spine surgery. These systems offer the potential advantages of increased accuracy of screw placement, decreased operative time, and decreased radiation exposure. However, there is a challenging learning curve, and various technical factors of these systems are continuously being reassessed to improve operative efficiency and to meet these goals. Maintaining clinical equipoise with established methods of screw placement, including freehand screw placement and various forms of navigation, will be important for further adoption of these systems in our field.

## Author Contributions

All authors listed have made a substantial, direct and intellectual contribution to the work, and approved it for publication.

## Conflict of Interest

JU receives consulting fees and royalties from NuVasive Medical, Inc., and is a consultant for Masonix, Inc., and SI Bone, Inc. JT receives consulting fees and royalties from NuVasive Medical, Inc. RP is the owner and founder of The Medical Memory, Inc. The remaining authors declare that the research was conducted in the absence of any commercial or financial relationships that could be construed as a potential conflict of interest.

## Abbreviations

CT: computed tomography.
